# Full-length transcriptome analysis identifies *SpRCC1* as a positive regulator of growth in *Spathiphyllum kochii*

**DOI:** 10.3389/fpls.2026.1755768

**Published:** 2026-02-13

**Authors:** Huifeng Lin, Xuanting Wang, Kezheng Xu, Zhi Ni, Lihong Li, Huiming Zhou, Fazhuang Lin, Xuming Xu, Jiajing Xiao, Nanyan Zhu, Danfeng Ge, Weifeng Xu

**Affiliations:** 1 College of Life Sciences, Center for Plant Water-use and Nutrition Regulation and College of JunCao Science and Ecology, Joint International Research Laboratory of Water and Nutrient in Crop, Fujian Agriculture and Forestry University, Fuzhou, China; 2 Sanming Academy of Agricultural Sciences, Sanming, Fujian, China; 3 FAFU-Dal Joint College (International College), Fujian Agriculture and Forestry University, Fuzhou, China; 4 Center for Agroforestry Mega Data Science, Haixia Institute of Science and Technology, Fujian Agriculture and Forestry University, Fuzhou, China

**Keywords:** full-length transcriptome, plant architecture, *Spathiphyllum kochii*, *SpRCC1*, WGCNA

## Abstract

Plant architecture is a critical agronomic trait that directly influences both the ornamental and commercial value of horticultural crops. *Spathiphyllum kochii*, a perennial evergreen herbaceous plant in the *Araceae* family, includes a naturally occurring dwarf mutant, Meibian’, which provides a valuable genetic resource for elucidating the molecular mechanisms controlling plant stature. In this study, we constructed a high-quality, full-length reference transcriptome based on the standard cultivar ‘Meijiu’ and its dwarf mutant ‘Meibian’ using PacBio Iso-Seq platforms. *De novo* assembly yielded 122,346 non-redundant high-confidence transcripts, including 94,809 predicted protein-coding sequences, of which 85,055 (89.71%) were successfully annotated in public databases. Phenotypically, ‘Meibian’ exhibits markedly reduced leaf, petiole, spathe, and spadix size compared to ‘Meijiu’. Transcriptome profiling across these four tissues identified 2,660 differentially expressed genes, with a global trend toward transcriptional repression in the dwarf mutant. Weighted Gene Co-expression Network Analysis revealed a key module significantly correlated with the dwarf phenotype, enriched in cytokinin-responsive genes and negative regulators of molecular functions. Among the hub genes, *Spathiphyllum kochii Regulator* of *Chromosome Condensation* 1 (*SpRCC1*) was identified as a potential key regulator. Full-length cloning confirmed a 2,303 bp coding sequence encoding a conserved RCC1 domain protein localized in the nucleus. Expression analysis showed significantly lower *SpRCC1* expression in ‘Meibian’, consistent with transcriptome data. Overexpression of *SpRCC1* in *Arabidopsis thaliana* resulted in increased leaf area, supporting its positive role in promoting growth. This study provides the first functional evidence linking *SpRCC1* to plant architecture regulation in *Spathiphyllum* and establishes a valuable transcriptomic resource for molecular breeding of compact ornamental cultivars.

## 1 Introduction

The *Araceae* family is a diverse group of monocotyledonous flowering plants, many of which are cultivated globally for ornamental purposes due to their striking foliage and distinctive inflorescence structures (Henriquez et al., 2014). Among these, *Spathiphyllum kochii* (*S. kochii*) stands out as a widely cultivated evergreen ornamental foliage plant, valued for its graceful white spathes, prolonged flowering period, lustrous green leaves, and well-documented air-purifying capabilities that enhance indoor environmental quality. In recent years, consumer preferences and urban living trends have driven an increased demand for compact and dwarf ornamental varieties, including *S. kochii*, as smaller plant forms are more suitable for confined indoor spaces and vertical gardens (Jain and Spencer, 2006; De, 2017; Malakar et al., 2025). Of particular interest is the naturally occurring dwarf species *Spathiphyllum pygmaeum*, which attains a modest height of only 10–15 cm and serves as a valuable genetic resource for breeding programs aimed at developing new dwarf cultivars with desirable ornamental and physiological traits (Bogner, 2011).

Plant architecture, determined by traits such as plant height, internode length, and branching pattern, is a fundamental agronomic characteristic that influences the visual and commercial value of ornamental plants, as well as their adaptability, stress tolerance and cultivation efficiency (Wang and Li, 2008; Teichmann and Muhr, 2015). Dwarf phenotypes have been well characterized across a range of species, including *Oryza sativa* (rice), *Zea mays* (maize), and *Arabidopsis thaliana*, where they are often associated with enhanced lodging resistance, reduced resource requirements, and improved appearance (Qin et al., 2007; Gao et al., 2009; Jiang et al., 2013; Lan et al., 2020). These traits are commonly governed by complex interactions between meristem activity, cell elongation, and phytohormone signaling pathways, most notably those involving gibberellins (GA), brassinosteroids (BR), and auxin (Guo et al., 2020; Berry and Argueso, 2022; Huang et al., 2022). Functional genomics studies in crops have identified numerous regulatory genes that modulate plant architecture through hormonal networks or chromatin-level transcriptional control. Recent advances in gene-editing technologies, such as CRISPR/Cas9, have further enabled precise manipulation of such genes, accelerating the rational design of optimized plant ideotypes with improved growth, resilience, and ornamental appeal (Huang et al., 2021). However, in the *Araceae* family, the molecular mechanisms underlying dwarfism remains largely unexplored. While dwarfing genes have been well characterized in model and crop species, few regulatory elements have been described in *Spathiphyllum*. The only reported candidate gene to date is *SpGH9A3*, which encodes a cellulase and has been associated with reduced leaf size and overall growth in the cultivar ‘Mojo’ (Yang et al., 2024).

In the absence of reference genome, transcriptome sequencing has become an vital tool for discovering genes and profiling their expression in non-model species. This technology enables the characterization of transcriptional activity at a genome-wide scale and provides critical insights into the molecular mechanisms underlying growth and developmental traits. It has been successfully applied to diverse plant taxa—including soybean, grape, lily, and maize—to elucidate genes associated with growth regulation, stress adaptation, and metabolic pathways (Wang et al., 2016, 2018; Ban et al., 2019; Howlader et al., 2020). Weighted Gene Co-expression Network Analysis (WGCNA) has further enhanced transcriptomic studies by enabling the identification of modules of co-expressed genes and hub regulators that coordinate complex biological processes, including plant height regulation, organ morphogenesis, and stress responses (Higashi and Saito, 2013; Qian et al., 2025). However, the lack of a high-quality reference genome in *S. kochii* has hindered comprehensive transcript annotation and reliable differential expression analysis. Recent advancements in third generation sequencing platforms, particularly the PacBio Iso-Seq system, have enabled the generation of high-fidelity, full-length cDNA sequences without the need for genome assembly (Gonzalez-Garay, 2015). This approach captures complete transcript isoforms, encompassing both coding and non-coding RNAs as well as alternative splicing events, thus offering a robust foundation for functional genomics and molecular breeding in *Spathiphyllum* and other non-model ornamental species.

In this study, we constructed the first full-length transcriptome of *S. kochii* using the PacBio Iso-Seq platform, based on two phenotypically contrasting cultivars: the standard medium-sized cultivar ‘Meijiu’ and its naturally occurring dwarf mutant ‘Meibian’. To capture a comprehensive expression profile, we sampled four representative tissues—leaf, petiole, spathe, and spadix—from both cultivars for comparative transcriptomic analysis. Through WGCNA, we identified a gene co-expression module that showed a strong correlation with the dwarf phenotype and pinpointed several hub genes potentially involved in plant architectural regulation. Among these, we discovered *SpRCC1* (*Spathiphyllum Regulator of Chromosome Condensation 1*), a nucleus-localized gene that displayed consistently reduced expression in the dwarf mutant across all tissues examined. Quantitative RT-PCR analysis further validated the differential expression of *SpRCC1*, and its heterologous overexpression in *Arabidopsis thaliana* resulted in increased larger leaves, supporting the hypothesis that downregulation of *SpRCC1* contributes to the compact growth habit of the dwarf mutant. Collectively, this study provides a comprehensive reference transcriptome for *S. kochii* and offers insights into the transcriptional networks associated with dwarfism. These findings establish a valuable molecular foundation for future breeding programs, enabling the development of compact *Spathiphyllum* cultivars through gene-targeted improvement strategies.

## 2 Materials and methods

### 2.1 Sample collection and RNA extraction

**Figure 1:**
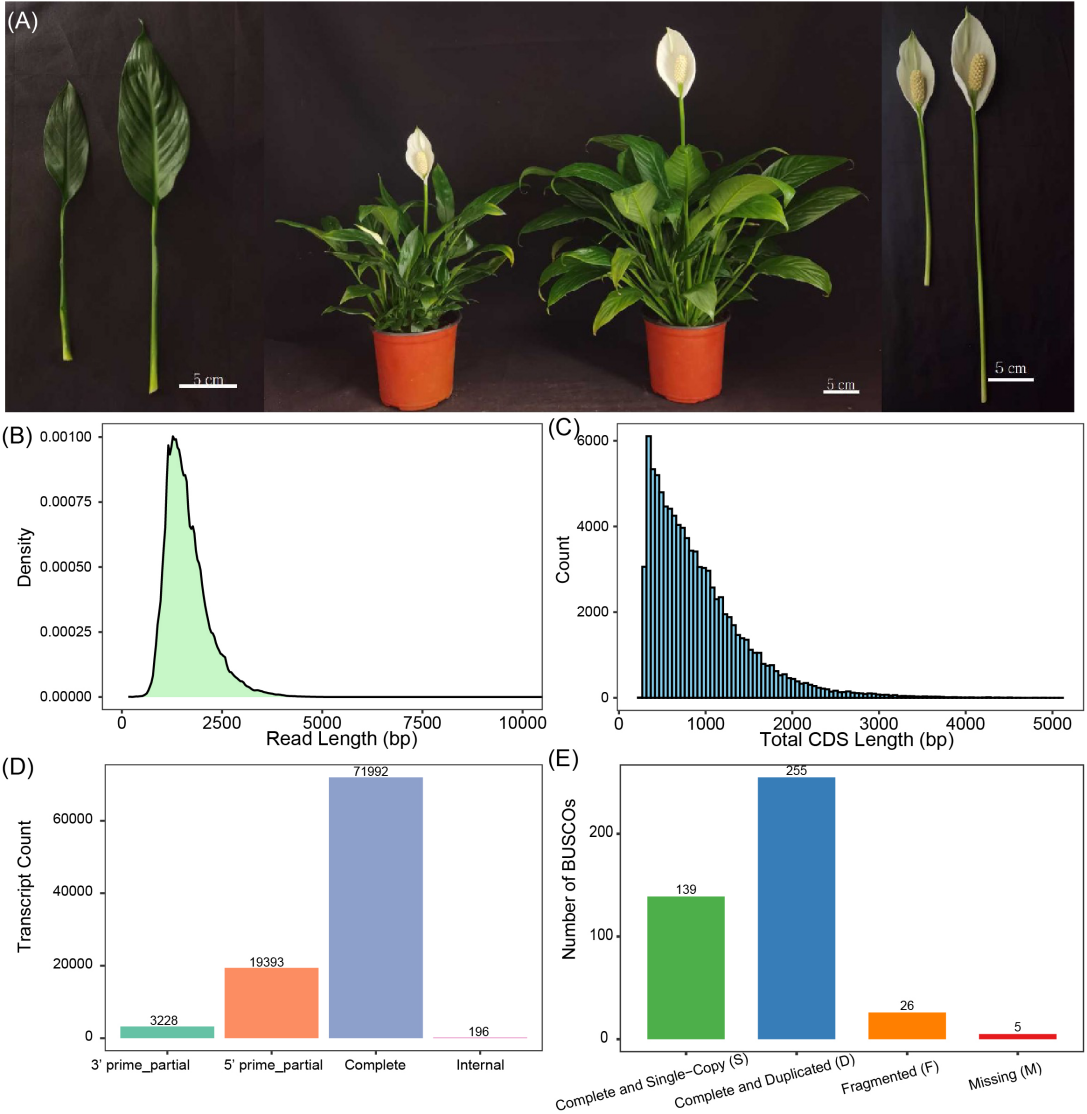
Overview of full-length transcriptome sequencing and annotation in *S. kochii*. **(A)** Morphological traits of the cultivar ‘Meijiu’ (right) and the dwarf mutant ‘Meibian’ (left). From left to right: comparison of leaf, whole-plant architecture, spathe and spadix morphology. Scale bars = 5 cm. **(B)** Read length 224 distribution of Circular Consensus Sequencing reads. **(C)** Distribution of coding sequence lengths for the 122,346 non-redundant reference transcripts. **(D)** Distribution of the competence of Open Reading Frame of the 94,809 transcripts. **(E)** The transcriptome completeness is evaluated via BUSCO assessment.

All plant materials were cultivated in the *Spathiphyllum* germplasm repository at Sanming Academy of Agricultural Sciences (Fujian, China). Two cultivars, Meijiu’ and its natural dwarf mutant ‘Meibian’, were sampled at the initial flowering stage under standardized greenhouse conditions (**Figure 1A**; **Supplementary Figure S1**). Four representative tissues—leaf (L), petiole (P), spathe (bract, B), and spadix (S)—were collected from each plant, with three biological replicates per tissue. The samples were immediately frozen in liquid nitrogen and stored at –80 °C for further experiments. Total RNA was extracted using TRIzol reagent (Invitrogen, USA), and RNA quality was assessed using NanoDrop spectrophotometry (Thermo Scientific, Waltham, MA, United States), and Agilent Bioanalyzer 2100 (Agilent Technologies, Santa Clara, CA, United States). To conduct full-length transcriptome sequencing, RNA samples were mixed together from the four tissues of both cultivars.

### 2.2 Full-length transcriptome sequencing and assembly

A total of 2 µg RNA was used to construct SMRTbell libraries with the SMRTbell^®^ Express Template Prep Kit 2.0 (PacBio), following the Iso-Seq Express protocol. Sequencing was performed on the PacBio Sequel II platform (Berry Genomics Co., Ltd., Beijing, China) (Gonzalez-Garay, 2015). Sequence data were processed using the SMRT Analysis software (ISOseq, version 4.2.0). Raw reads were processed using SMRTLink v10.1 to generate HiFi reads (parameters: --min-passes=3, --min-rq=0.99). Full-length non-chimeric (FLNC) reads were defined as sequences containing both 5′ and 3′ primers and poly(A) tails, excluding internal primers. FLNC reads were clustered to generate high-quality consensus isoforms. Redundancy was removed using CD-HIT (version 4.8.1; -c 0.90 -s 0.85), resulting in a non-redundant transcript set. Coding sequences (CDSs) were predicted using TransDecoder, with an ORF length cutoff of 100 amino acids. Functional annotation was conducted against the NR (NCBI non-redundant protein database), Swiss-Prot (manually curated protein database), KOG (EuKaryotic Orthologous Groups), KEGG, InterPro, and Gene Ontology (GO) databases using DIAMOND BLASTX (E-value < 1e–10).

### 2.3 Expression profile analysis of dwarf phenotype in *S. kochii*

Transcriptome profiling of the four tissues from both genotypes was performed using Illumina NovaSeq 6000 (Genedenovo Biotechnology Co., Ltd., Guangzhou, China), yielding 24 paired-end libraries (150 bp). Raw sequencing reads were processed with fastp (version 0.23.4) for quality control and adaptor trimming to generate high-quality clean reads (Chen, 2023). PacBio full-length transcript sequences were used as reference transcripts. Gene expression levels were quantified with Bowtie2 (version 2.5.1) for alignment Clean reads were mapped to the PacBio-derived reference transcriptome using Bowtie2 (version 2.5.1), and expression levels were calculated with RSEM, normalized as fragments per kilobase of transcript per million mapped reads (FPKM) (Langmead and Salzberg, 2012). Differentially expressed genes (DEGs) were identified using DESeq2 with thresholds of |log_2_(fold change)| ≥ 1 and false discovery rate (FDR) < 0.05 (Love et al., 2014). Functional enrichment analyses were performed using the clusterProfiler and topGO packages (Alexa and Rahnenführer, 2009; Wu et al., 2021). The final visualization was generated in R (version 4.4.0). WGCNA was applied to the expression matrix of all DEGs using the WGCNA R package (Langfelder and Horvath, 2008). A soft threshold power was selected based on scale-free topology criteria (R² > 0.85). Module–trait relationships were calculated via Pearson correlation between module eigengenes and phenotype traits. Hub genes were defined by high module membership (MM > 0.8) and gene significance (GS > 0.6).

### 2.4 Gene cloning and subcellular localization of *SpRCC1*

Poly(A)+ RNAs were isolated and subjected to 5′ and 3′ RACE using the GeneRacer™ Kit (Invitrogen) to obtain the full-length coding sequence of *SpRCC1*. For 3′ RACE, poly(A)+ RNAs were reverse transcribed with the GeneRacer oligo(dT) primer, and the resulting cDNAs were amplified using the GeneRacer 3′ outer/inner primers and gene-specific primers. The 5′ end of *SpRCC1* was amplified using the 5′ RACE System. Amplified fragments were purified, cloned, and sequenced. Primer sequences used in RACE are listed in **Supplementary Table 1**. The CDS of *SpRCC1* was cloned into the pBWA(V)H2STMVΩ-egfp vector, and the constructed plasmid was transformed into *Agrobacterium tumefaciens* GV3101 and transiently expressed in *Nicotiana benthamiana*. The fluorescence signal was observed using a LSM 880 microscope (Zeiss, Germany) for subcellular localization analysis, with NLS-RFP used as a nuclear marker.

### 2.5 qRT-PCR validation of *SpRCC1*

First-strand cDNA was synthesized using the HiScript^®^ Reverse Transcriptase kit according to the manufacturer’s protocol. The resulting cDNA was diluted 10-fold and used as a template for quantitative real-time PCR (qRT-PCR) with ChamQ Universal SYBR^®^ qPCR Master Mix. *Actin* was used as an internal reference gene. Relative expression was calculated using the 2^–ΔΔCt method. Primer sequences are listed in **Supplementary Table 1**.

### 2.6 Stable transformation in *Arabidopsis thaliana*

The full-length *SpRCC1* CDS was inserted into the binary vector pBWA(V)BS and transformed into *Arabidopsis thaliana Col-0* via the floral dip method. Transformed plants were grown under a 16 h light/8 h dark photoperiod at 25 °C, and mature T1 seeds were harvested and dried with silica gel. Following surface sterilization, seeds were selected on ½ MS medium with 15 mg L^-1^ basta. Resistant seedlings were transferred to soil and grown under the same photoperiod and temperature conditions. Genomic DNA was extracted from young leaf tissues using the CTAB method. Transgene integration was verified by PCR using gene-specific and vector-specific primers (**Supplementary Table 1**). Segregation analysis of T1 progeny confirmed a 3:1 ratio of resistant to sensitive seedlings, indicative of single-locus insertion. Independent T2 lines with single-copy insertions were further propagated, and homozygous T2 lines were identified by PCR-based genotyping. At eight weeks post-germination, leaf morphology was examined, and differences in leaf area were recorded between over-expression lines and wild-type (WT, *Col-0*). For expression validation, rosette leaves were harvested from both genotypes, flash-frozen in liquid nitrogen, and subjected to qRT-PCR to assess SpRCC1 transcript abundance.

## 3 Results

### 3.1 Construction of a high-quality full-length transcriptome reference for *S. kochii*

The *Spathiphyllum* cultivar ‘Meibian’ (MBZ) is a naturally occurring mutant derived from the standard cultivar ‘Meijiu’ (BZ). Compared to ‘Meijiu’, Meibian’ exhibits markedly smaller vegetative and reproductive organs, resulting in a compact overall growth habit (**Figure 1A**). To elucidate the molecular basis of this architectural variation, we performed full-length transcriptome sequencing of *S. kochii*. A total of 10,081,745 circular consensus sequencing (CCS) reads were generated, yielding approximately 15.65 Gb of high-quality data. Most of the CCS reads ranged from 1,000 to 2,000 base pairs (bp) in length, consistent with expected full-length cDNA distributions (**Figure 1B**; **Supplementary Table 2**). From these data, 523,557 high-confidence transcripts were identified. Transcript length distribution analysis revealed that the majority also fell within the 1,000–2,000 bp range (**Figure 1C**), further supporting the integrity of the sequencing data. After removing redundancy, a total of 122,346 non-redundant transcript isoforms were retained to establish the first full-length reference transcriptome of *S. kochii*.

Among the assembled transcripts, 94,809 transcripts (77.5%) were predicted to possess protein-coding potential, as determined by CDS analysis (**Figure 1D**). Notably, 71,992 transcripts (75.9% of coding transcripts) contained complete open reading frames (ORFs), including both canonical start and stop codons, highlighting the high structural accuracy of the assembled transcriptome. The analysis revealed that 92.5% of conserved core plant genes were completely recovered, including 139 single-copy and 255 duplicated orthologs, with only 1.2% of orthologs classified as missing (**Figure 1E**). These results confirm that the full-length transcriptome of *S. kochii* is highly complete, accurate, and suitable as a reference resource for future functional, regulatory, and comparative genomic analyses in this species.

**Figure 2:**
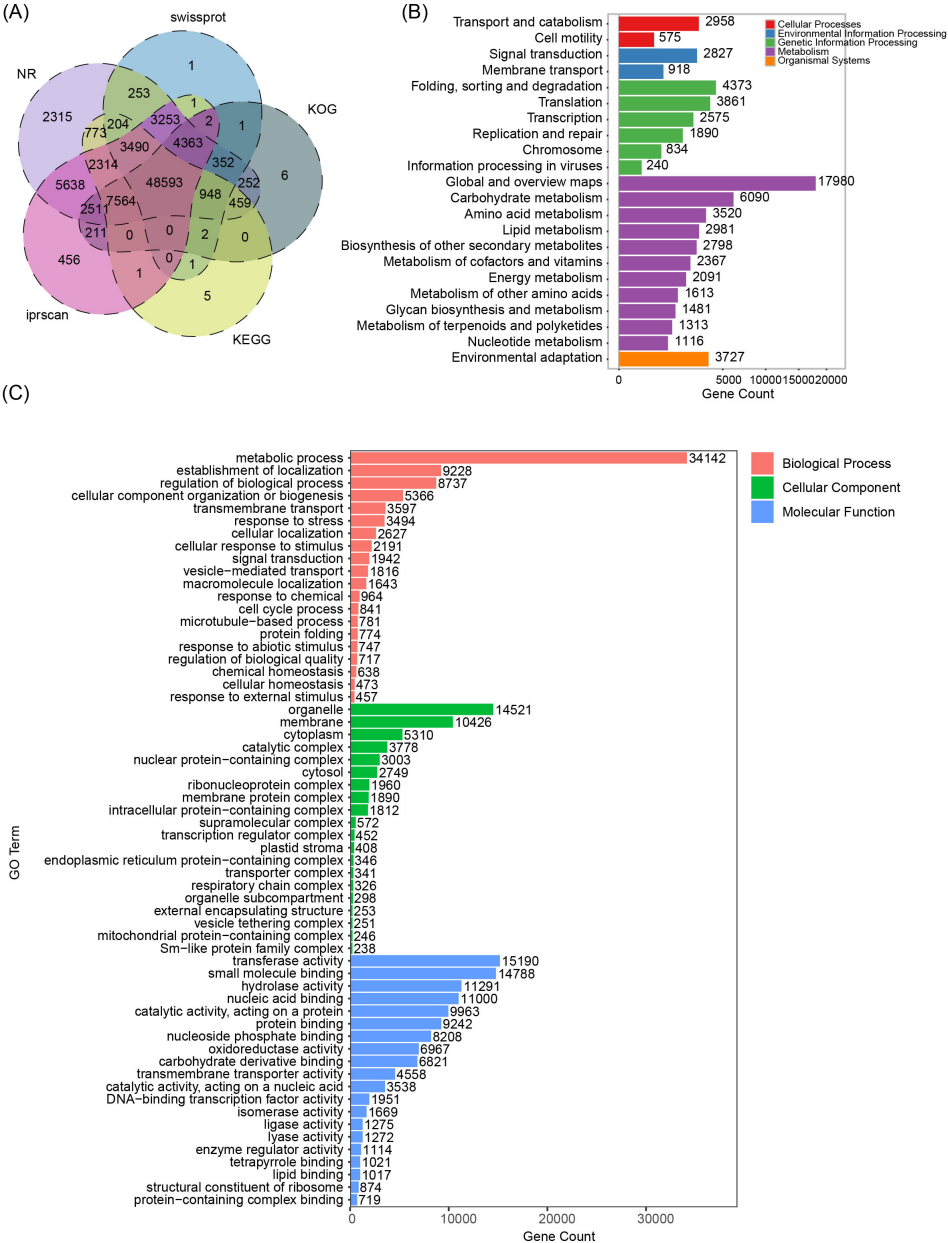
Functional annotation of *S. kochii* transcripts across five major databases. **(A)** Venn diagram showing the distribution of annotated transcripts in NR, SwissProt, KOG, KEGG, and InterProScan databases. Functional classification of annotated transcripts in *S. kochii*. **(B)** Gene Ontology (GO) classification of annotated transcripts into the categories of Biological Process (red), Cellular Component (blue), and Molecular Function (green). Bars represent the number of genes assigned to each GO term. **(C)** KEGG functional classification of annotated transcripts across five major categories: Cellular Processes (red), Environmental Information Processing (blue), Genetic Information Processing (green), Metabolism (purple), and Organismal Systems (orange). Bars indicate the number of genes mapped to each pathway.

Functional annotation was performed on the 94,809 non-redundant protein-coding transcripts derived from the full-length transcriptome assembly. A total of 85,055 transcripts (89.71%) were successfully annotated in at least one public database, underscoring the reliability and completeness of the assembled transcriptome. Notably, 48,594 transcripts were simultaneously annotated across all five databases used, reflecting the robustness of the annotation strategy and the comprehensive functional representation of the *S. kochii* transcriptome (**Figure 2A**; **Supplementary Table 3**). Gene Ontology (GO) classification assigned functional terms to 58,856 transcripts (**Figure 2B**). Within the biological process category, 40,662 transcripts were annotated, with the majority involved in metabolic processes (34,142 transcripts), followed by localization-related processes (9,228 transcripts) and regulation of biological processes (8,737 transcripts). In the cellular component category, 32,890 transcripts were annotated, predominantly associated with organelles (14,521 transcripts) and membranes (10,462 transcripts), indicating broad transcript coverage across subcellular compartments. For the molecular function category, 49,087 transcripts were annotated, with the most enriched functions including transferase activity (15,190 transcripts) and small molecule binding (14,788 transcripts), reflecting the transcriptome’s strong representation of enzymatic and regulatory functions.

A total of 64,354 transcripts were successfully annotated with KEGG pathway information and mapped to five major functional categories (**Figure 2C**). The largest category was ‘Metabolism’, encompassing a broad range of primary metabolic processes. Within this group, transcripts were particularly enriched in pathways related to carbohydrate metabolism (6,090 transcripts), amino acid metabolism (3,528 transcripts), and lipid metabolism (2,981 transcripts), highlighting the essential role of primary metabolism in sustaining cellular growth and developmental processes in *S. kochii*. The Genetic Information Processing category included transcripts involved in translation (3,861 transcripts), transcription (2,575 transcripts), and replication and repair (1,890 transcripts), indicating an active landscape of gene expression and genomic maintenance. The Environmental Information Processing category featured genes associated with signal transduction (2,827 transcripts) and membrane transport (918 transcripts), reflecting the plant’s capacity to perceive and respond to environmental stimuli. In the ‘Organismal Systems’ category, a notable number of transcripts (3,727) were assigned to environmental adaptation, suggesting that *S. kochii* possesses transcriptional programs responsive to abiotic or biotic environmental cues.

### 3.2 Expression profiles difference between the two phenotypes in *S. kochii*

To elucidate the molecular mechanisms underlying the dwarf phenotype in *S. kochii*, we performed comparative transcriptomic profiling of four representative tissues from two cultivars (**Figure 1A**). Each RNA-seq library produced approximately 62.3 million raw paired-end reads, yielding a total of 191.22 Gb of raw data across all samples (**Supplementary Table 4**). After quality control and filtering, 188.82 Gb of high-quality clean data were retained for downstream analysis. Transcript abundance was quantified using the previously constructed full-length reference transcriptome.

**Figure 3:**
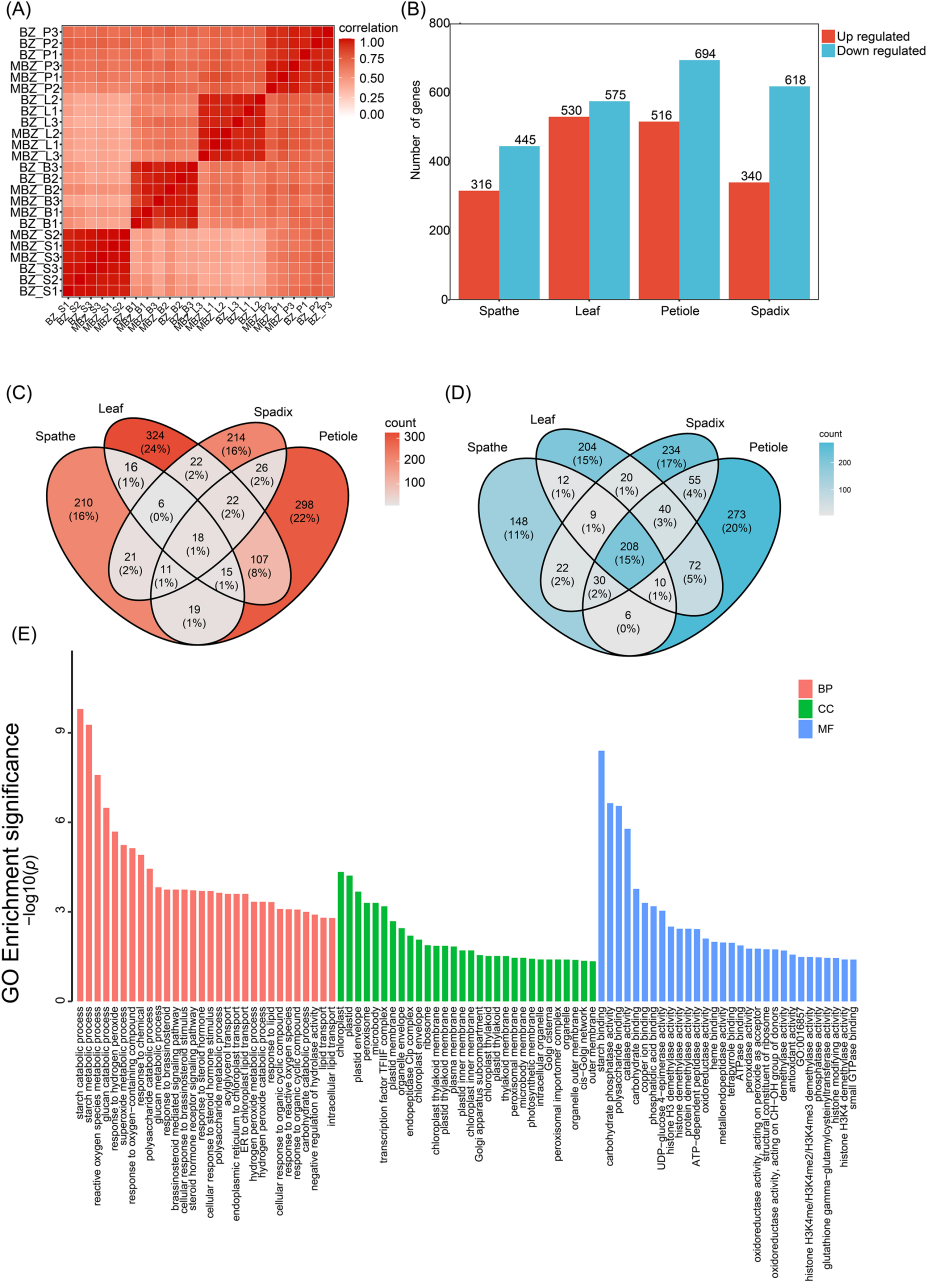
Transcriptome-wide differential expression analysis between the standard cultivar ‘Meijiu’ (BZ) and the dwarf mutant ‘Meibian’ (MBZ) across four tissues. **(A)** Heatmap of Pearson correlation coefficients among all samples. **(B)** Bar plot showing the number of significantly upregulated (red) and downregulated (blue) DEGs in spathe (BZ_B vs. MBZ_B), leaf (BZ_L vs. MBZ_L), petiole (BZ_P vs. MBZ_P), and spadix (BZ_S vs. MBZ_S). **(C)** Venn diagram of upregulated DEGs across the four tissue comparisons, illustrating tissue-specific and shared expression patterns. **(D)** Venn diagram of downregulated DEGs across tissues, highlighting a core set of 208 genes consistently repressed in all organs of the dwarf mutant. **(E)** GO enrichment analysis of the 208 commonly downregulated DEGs across all tissues. Bar plot displays significantly enriched GO terms across three categories: Biological Process (BP, red), Cellular Component (CC, green), and Molecular Function (MF, blue). The x-axis represents –log_10_(p-value), and numbers at the end of each bar indicate the number of DEGs annotated to each term.

Pearson correlation analysis demonstrated strong consistency among biological replicates for each tissue and genotype (**Figure 3A**). Differential expression analysis revealed a total of 2,660 significantly differentially expressed genes (DEGs) between ‘Meijiu’ and ‘Meibian’ across all tissues, reflecting extensive transcriptional reprogramming associated with the dwarf phenotype. Specifically, we identified 761, 1,105, 1,210, and 958 DEGs in the spathe, leaf, petiole, and spadix, respectively (**Figure 3B**). In all examined tissues, the number of downregulated genes exceeded upregulated genes, indicating a global trend of transcriptional repression in the dwarf mutant. This widespread reduction in gene expression likely contributes to its smaller organ size and compact growth morphology.

DEG overlap analysis revealed both tissue-specific and shared transcriptional expression changes between ‘Meijiu’ and ‘Meibian’. Among the upregulated DEGs, most were uniquely expressed in specific tissues: 324 in the leaf, 298 in the petiole, 214 in the spadix, and 210 in the spathe. Only 18 genes (1%) were consistently upregulated across all tissues (**Figure 3C**). In contrast, downregulated genes exhibited greater overlap, with 208 transcripts consistently repressed in all tissues examined (**Figure 3D**), suggesting the presence of a core transcriptional repression module associated with the dwarf phenotype of ‘Meibian’.

GO enrichment analysis of DEGs revealed organ-specific functional disruptions linked to growth inhibition. In the leaf, downregulated DEGs were significantly enriched in photosynthesis (GO:0015979) and response to BR (GO:0009741), suggesting that reductions in energy production and hormone signaling may be associated with leaf expansion. In the petiole, DEGs were enriched in BR homeostasis (GO:0010268), BR biosynthetic process (GO:0016132), and phytosteroid biosynthetic process (GO:0016129), indicating altered expression of genes involved in hormone-related pathways associated with cell elongation. A similar pattern was observed in the spadix, with suppression of genes involved in BR biosynthesis and response pathways, further supporting the hypothesis that impaired BR metabolism is a major factor contributing to reduced reproductive organ size. In the spathe, downregulated genes were predominantly associated with flavonoid biosynthesis (GO:0009813) and response to red or far-red light (GO:0009639), implying altered secondary metabolism and photomorphogenic signaling.

To further explore the functions of the 208 genes consistently downregulated across all tissues, GO enrichment analysis was performed (**Figure 3E**). In the biological process category, enriched terms included starch catabolic process, glucan catabolic process, response to BR, BR-mediated signaling pathway, and response to steroid hormone, representing transcriptional reprogramming related to developmental regulation. In the cellular component category, significant enrichment was observed for chloroplast, plastid, and thylakoid membrane-related terms, indicating impaired photosynthetic capacity and plastidial function in the dwarf mutant. In the molecular function category, enriched terms included carbohydrate binding, hydrolase activity, oxidoreductase activity, and notably, histone demethylase activity—implying potential involvement of epigenetic modifications in the transcriptional repression of growth-related genes.

Collectively, these findings collectively indicate that the dwarf phenotype in *S. kochii* arises from coordinated repression of key pathways controlling vegetative and reproductive development. The systemic downregulation of genes related to BR signaling, energy metabolism, and chloroplast function, coupled with reduced activity of regulatory enzymes such as histone demethylases, suggests a multi-layered regulatory mechanism underlying the compact phenotype of ‘Meibian’. This convergent repression likely defines a core transcriptional program that restricts cell expansion and organ growth, giving rise to the compact architecture of ‘Meibian’.

### 3.3 Identification of hub genes associated with the dwarfism phenotypes

**Figure 4:**
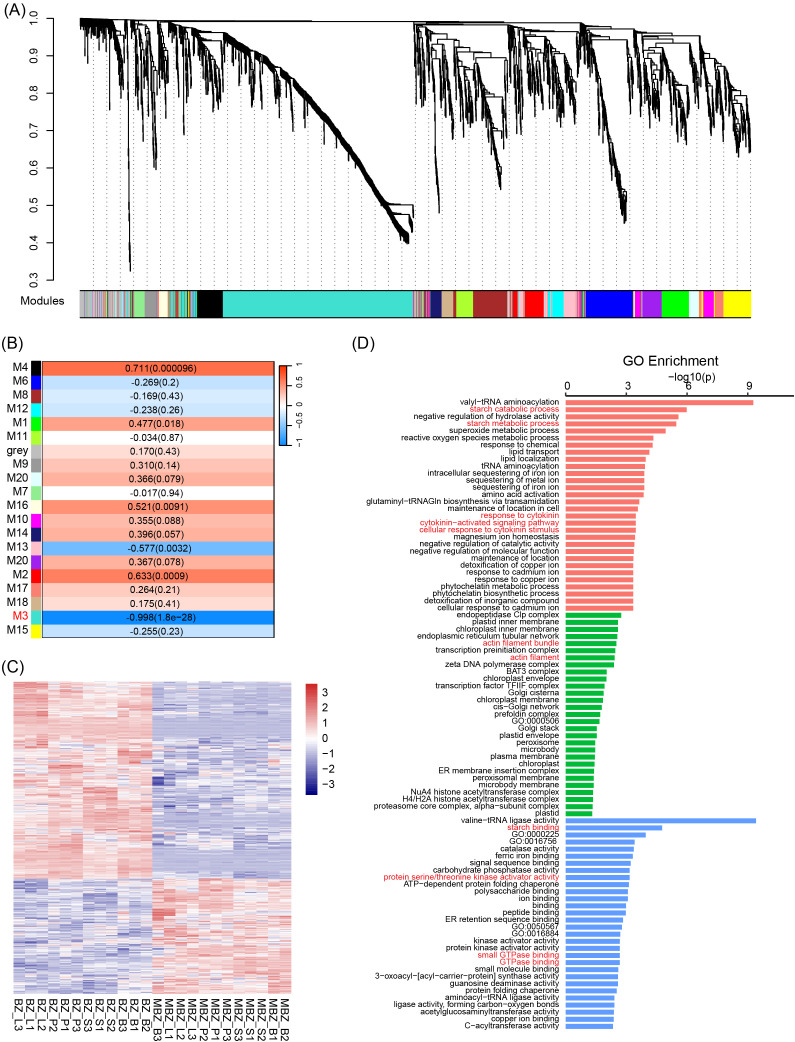
Weighted gene co-expression network analysis (WGCNA) of *S. kochii* transcripts. **(A)** Hierarchical clustering dendrogram of all differentially expressed genes (DEGs) across all tissues. Genes with similar expression profiles were grouped into 19 distinct co-expression modules, each represented by a unique color. **(B)** Module–trait correlation heatmap. Each row corresponds to a module eigengene and each column to a phenotype. Numbers in each cell represent the Pearson correlation coefficient and the corresponding p-value. **(C)** Heatmap of expression profiles of genes in the M3 module across 24 samples (3 biological replicates each for four tissues in both ‘Meijiu’ and ‘Meibian’). Expression values are scaled by Z-score per gene. **(D)** Gene Ontology (GO) enrichment analysis of M3 module genes. Bar plot displays significantly enriched GO terms across three categories: Biological Process (BP, red), Cellular Component (CC, green), and Molecular Function (MF, blue). The x-axis represents –log_10_(p-value), and numbers at the end of each bar indicate the number of DEGs annotated to each term.

To further explore the regulatory networks associated with the dwarf phenotype in *S. kochii*, a weighted gene co-expression network analysis (WGCNA) was performed based on the expression profiles of the 2,660 DEGs identified across the four tissues. The analysis identified 19 distinct co-expression modules, each representing a cluster of genes with highly correlated expression patterns (**Figure 4A**). Among these, module M3 exhibited a strong negative correlation with the dwarf genotype (Pearson’s r = –0.998, *p* = 1.81 × 10^-28^), indicating that genes within this module are tightly associated with plant size and growth regulation (**Figure 4B**). Module M3 contained 843 genes showing highly coordinated expression patterns, with markedly different expression levels between the standard cultivar ‘Meijiu’ and the dwarf mutant ‘Meibian’ (**Figure 4C**). Hub genes within M3 were identified based on high module membership and gene significance, reflecting their prominent positions within the co-expression network. Heatmap analysis revealed that the majority of these hub genes were consistently downregulated across all examined tissues in ‘Meibian’ (**Supplementary Figure S2**), suggesting a coordinated shift in the expression of genes linked to growth- and development-related processes. This widespread downregulation highlights transcriptional differences in co-expressed gene networks associated with the compact growth phenotype of the dwarf mutant.

Several representative hub genes in M3 have been previously implicated in plant growth–related pathways. *SpBSK3* (BR Signaling Kinase 3) is a component of the brassinosteroid signaling pathway and has been reported to participate in cell elongation and organ growth (Zolkiewicz and Gruszka, 2022). *SpHTH* (HOT-HEAD-like protein) has been implicated in lipid metabolic processes associated with epidermal integrity and organ development (Krolikowski et al., 2003). *SpJMJ25* (Jumonji domain-containing protein 25) encodes a histone demethylase implicated in the epigenetic regulation of growth-related genes (Yamaguchi, 2021), while *SpBBX19* (B-box zinc finger protein 19) functions as a light-responsive transcription factor that modulates photomorphogenesis and internode elongation (Gao et al., 2024). Notably, *SpRCC1* (*Spathiphyllum kochii Regulator of Chromosome Condensation 1*) is predicted to be nucleus-localized and involved in chromatin organization and cell cycle regulation. he interaction between RCC1 and small GTPases has been shown to play an essential role in plant development and stress responses, suggesting that *SpRCC1* may act as a key integrator of chromatin remodeling and signal transduction during growth regulation. Furthermore, GO enrichment analysis of all genes within module M3 revealed significant overrepresentation of biological processes such as response to cytokinin (GO:0009735), negative regulation of catalytic activity (GO:0043086), and negative regulation of molecular function (GO:0044092) (**Figure 4D**). These results indicate that M3 integrates hormone-responsive signaling with transcriptional repression mechanisms. Together, these transcriptional enrichments suggest coordinated hormone-responsive pathways involving cytokinins, BR, and auxin that may contribute to growth and morphological variation in the dwarf mutant.

### 3.4 Cloning and functional characterization of *SpRCC1*

**Figure 5:**
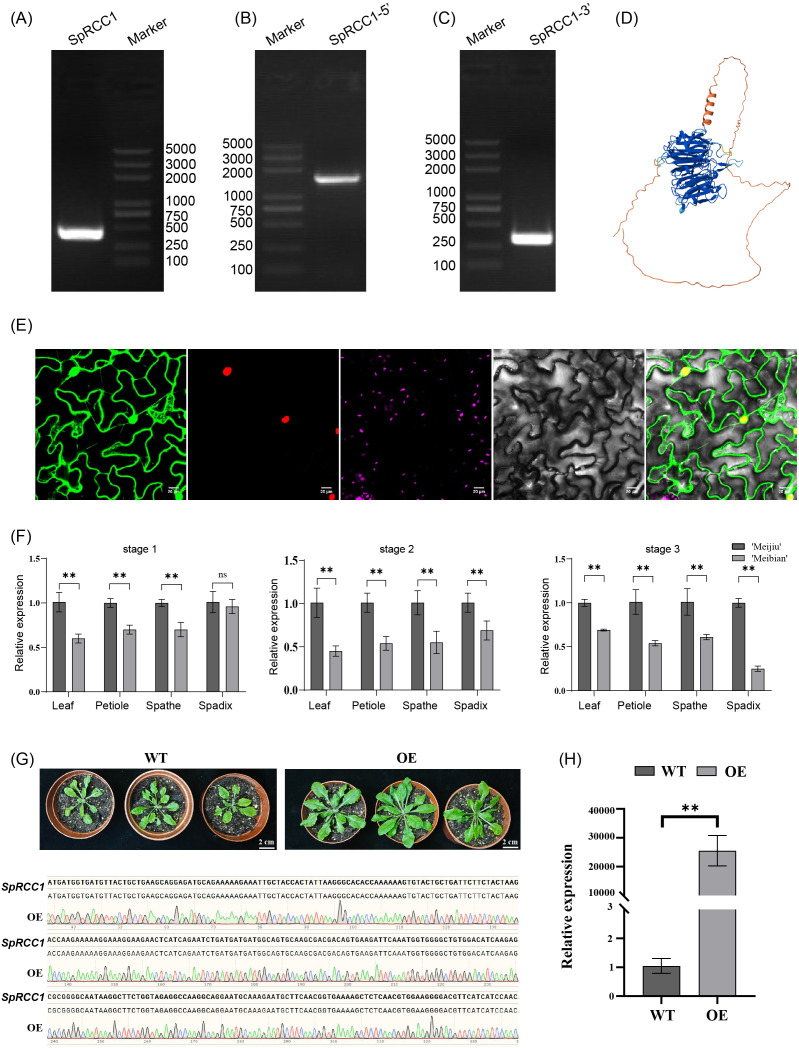
Molecular characterization, structural prediction, subcellular localization, and expression analysis of *SpRCC1* in *S. kochii*. **(A–C)** Amplification of the full-length coding sequence of *SpRCC1* using RACE, showing products from **(A)** full-length, **(B)** 5′ end, and **(C)** 3′ end. **(D)** Predicted 3D structure of SpRCC1 generated by AlphaFold2, reveals the canonical seven-bladed β-propeller fold characteristic. **(E)** Subcellular localization of SpRCC1. From left to right: GFP fluorescence (green, left panel), nuclear localization signal (RFP, red), chromatin dye (Hoechst, purple), bright-field image, and merged channels. Scale bars = 20 μm. **(F)** Relative expression levels of *SpRCC1* in leaf, petiole, spathe, and spadix tissues across three developmental stages of the cultivar ‘Meijiu’ and the dwarf mutant ‘Meibian’, including the initial emergence stage (stage 1), initial flowering stage (stage 2), and mature stage (stage 3). Expression levels were normalized to the lowest-expressing sample within each tissue. Data are presented as mean ± SD of three biological replicates. Significant differences were determined using Student’s t-test (**p < 0.01). **(G)** The leaf phenotype of 8-week-old seedlings of wild-type (WT), and *SpRCC1* over-expression lines (OE). **(H)** Relative expression analysis of *SpRCC1* in wild-type (WT) and overexpression (OE) *Arabidopsis thaliana* lines by qRT-PCR. Actin was used as the internal reference gene. *SpRCC1* expression was undetectable in WT plants (assigned Ct = 40 for calculation). Error bars represent mean ± SD (n = 3), reflecting minor variation in reference gene Ct values across replicates. p < 0.01 by Student’s t-test.

To elucidate the functional role of *SpRCC1* in regulating plant architecture, the full-length cDNA sequence was obtained using RACE. The obtained cDNA sequence was 2,303 bp in length and encoded a 556–amino acid protein containing a conserved RCC1 domain (Pfam: PF00415), a characteristic feature of the Regulator of Chromosome Condensation 1 (RCC1) protein family in plants (**Figures 5A–C**). Structural prediction using AlphaFold2 revealed that SpRCC1 adopts a canonical seven-bladed β-propeller fold, a hallmark structural feature of RCC1 proteins known to function in Ran GTPase signaling and nucleocytoplasmic transport (**Figure 5D**). The conservation of this structural motif suggests that *SpRCC1* likely retains biochemical functions related to chromatin organization and cell cycle regulation.

Subcellular localization analysis demonstrated that SpRCC1 is predominantly localized in the nucleus (**Figure 5E**). qRT-PCR analysis revealed that *SpRCC1* expression was significantly lower in the dwarf mutant ‘Meibian’ compared to the standard cultivar ‘Meijiu’ across all tested tissues and developmental stages (**Figure 5F**). This consistent downregulation supports the functional association of SpRCC1 with growth regulation.

Functional characterization through heterologous overexpression in *Arabidopsis thaliana* demonstrated a marked enhancement of vegetative growth, with transgenic plants exhibiting significantly increased leaf area compared to wild-type controls (**Figures 5G, H**; **Supplementary Figure S3**). These morphological changes support a positive regulatory role of SpRCC1 in promoting plant growth. The phenotypic concordance between *Arabidopsis* overexpression lines and the reduced expression of *SpRCC1* in the dwarf *Spathiphyllum* mutant further validates its functional role in determining plant size and architecture.

## 4 Discussions

*Spathiphyllum kochii* is a widely cultivated ornamental plant valued for its broad, glossy foliage and prolonged flowering period. In modern urban horticulture, compact and dwarf phenotypes are increasingly preferred due to their suitability for limited spaces, enhanced manageability, and commercial appeal (Malakar et al., 2025). However, the genomic and molecular understanding of plant architecture in *S. kochii* remains limited, primarily due to the absence of a reference genome and the lack of functional genomics tools. The identification of a naturally occurring dwarf mutant, ‘Meibian’, derived from the standard cultivar ‘Meijiu’, offers a valuable opportunity to investigate the genetic and regulatory mechanisms underlying dwarfism in this species.

This study represents the first comprehensive molecular analysis of dwarfism in *S. kochii*, integrating long-read PacBio Iso-Seq, short-read Illumina RNA-Seq, and gene co-expression network analysis. The construction of a high-quality full-length reference transcriptome, comprising 122,346 non-redundant transcripts and 94,809 protein-coding genes, fills a critical gap in molecular resources for this non-model species. With over 89% of transcripts successfully annotated, the dataset provides a robust platform for gene expression profiling, pathway enrichment, and candidate gene discovery—thereby laying a solid foundation for functional genomics and molecular breeding in *Spathiphyllum* and related members of the *Araceae*.

Comparative transcriptomic analysis between ‘Meijiu’ and ‘Meibian’ across four representative tissues (leaf, petiole, spathe, and spadix) identified 2,660 DEGs, with the majority being downregulated in the dwarf mutant. This pattern is consistent with findings in other species, where dwarfism is often associated with transcriptional suppression of genes involved in hormone biosynthesis, signal transduction, cell elongation, and meristem activity (Li et al., 2012; Lu et al., 2022; Ma et al., 2022; Fang et al., 2024). The widespread and coordinated downregulation observed in ‘Meibian’ suggests a systemic regulatory shift, rather than tissue-specific developmental defects.

WGCNA revealed a key co-expression module (M3) that was strongly associated with the dwarf phenotype. Functional enrichment of this module revealed significant associations with cytokinin response, chromatin organization, and negative regulation of molecular activity. These biological processes are central to growth control and organ development. The reduced expression of genes within this module reflects altered transcriptional patterns across hormone-associated and regulatory pathways in the dwarf mutant. Among the candidate genes in module M3, several are known regulators of growth-related pathways. Genes involved in BR signaling and auxin transport, such as those encoding BR signaling kinases and auxin efflux proteins, were significantly downregulated. These pathways are well-established mediators of cell expansion and elongation in diverse species (Zhang et al., 2010; Leyser, 2018; Ren et al., 2019). Additionally, genes associated with light signaling and photomorphogenesis, chromatin remodeling, and epigenetic modification were also suppressed, suggesting that plant architecture is influenced by a complex interplay between hormonal cues, environmental perception, and chromatin state (Wang et al., 2015; Matsuo et al., 2022).

One key gene identified was *SpRCC1* (Regulator of Chromosome Condensation 1), a conserved nuclear-localized factor with predicted roles in chromatin organization, nucleocytoplasmic transport, and cell cycle regulation (Dasso, 1993). RCC1 family proteins function as guanine nucleotide exchange factors for Ran GTPase, and in plants, have been implicated in developmental timing and stress responses (Li et al., 2022, 2024; Liu et al., 2024). In this study, *SpRCC1* was consistently downregulated in all tissues of the dwarf mutant and exhibited reduced expression throughout developmental stages. Structural modeling confirmed its conserved β-propeller fold, and subcellular localization experiments verified nuclear enrichment. Most notably, overexpression of *SpRCC1* in *Arabidopsis* resulted in significantly increased plant height and larger leaf size, functionally validating its role as a positive regulator of plant growth *in vivo*. This finding supports the hypothesis that reduced expression of *SpRCC1* in ‘Meibian’ contributes causally to the dwarf phenotype, likely through impaired chromatin regulation and diminished cell proliferation.

The global trend of transcriptional repression observed in ‘Meibian’ aligns with a broader pattern reported in other species, where growth-inhibitory states are characterized by reduced expression of genes involved in hormone metabolism, carbohydrate processing, and developmental signaling. The repression of *SpJMJ25*, a histone demethylase, further suggests that epigenetic constraints may underline reduced transcriptional activity in the dwarf mutant. The concurrent downregulation of BR-related genes and epigenetic regulators indicates possible feedback loops in which hormone imbalance and chromatin-mediated silencing reinforce each other to limit growth. Importantly, several hub genes within the M3 module were expressed consistently across all tissues examined, reinforcing the notion that systemic regulatory factors—not merely localized defects—underlie the dwarfism observed in ‘Meibian’. These findings provide mechanistic insight into how disruptions in coordinated transcriptional programs can reshape overall plant morphology. While this study advances our understanding of plant architecture regulation in *Spathiphyllum*, further investigation is needed to clarify the upstream signals triggering these transcriptional changes. Functional validation through gene knockout or overexpression, particularly of *SpRCC1*, BR signaling components, and chromatin modifiers, will be crucial to confirm their causal roles. Future studies incorporating hormone profiling and promoter activity assays may help determine whether changes in hormone biosynthesis or sensitivity contribute to the transcriptional differences observed in this module.

In conclusion, this study provides the first high-resolution transcriptomic framework for understanding plant architecture regulation in *Spathiphyllum kochii*. Through integrative sequencing and co-expression network analysis, we identified key pathways and regulators, particularly *SpRCC1*, that contribute to plant architectural variation. These insights not only advance the fundamental understanding of ornamental plant growth control but also provide valuable molecular targets for developing compact, space-efficient cultivars via biotechnological approaches.

## Data Availability

The datasets presented in this study can be found in online repositories. The names of the repository/repositories and accession number(s) can be found below: https://ngdc.cncb.ac.cn/gsa, CRA030630 and CRA030638.
