# Supplementary material for: Full-length transcriptome analysis identifies SpRCC1 as a positive regulator of growth in Spathiphyllum kochii
Source: Front Plant Sci. 2026 Feb 13;17:1755768. doi: 10.3389/fpls.2026.1755768 (PMC12946143; doi:10.3389/fpls.2026.1755768)
Supplement: Supplementary file 1 [file Table1.docx]

Supplementary Material

# Supplementary Figures and Tables

## Supplementary Figures


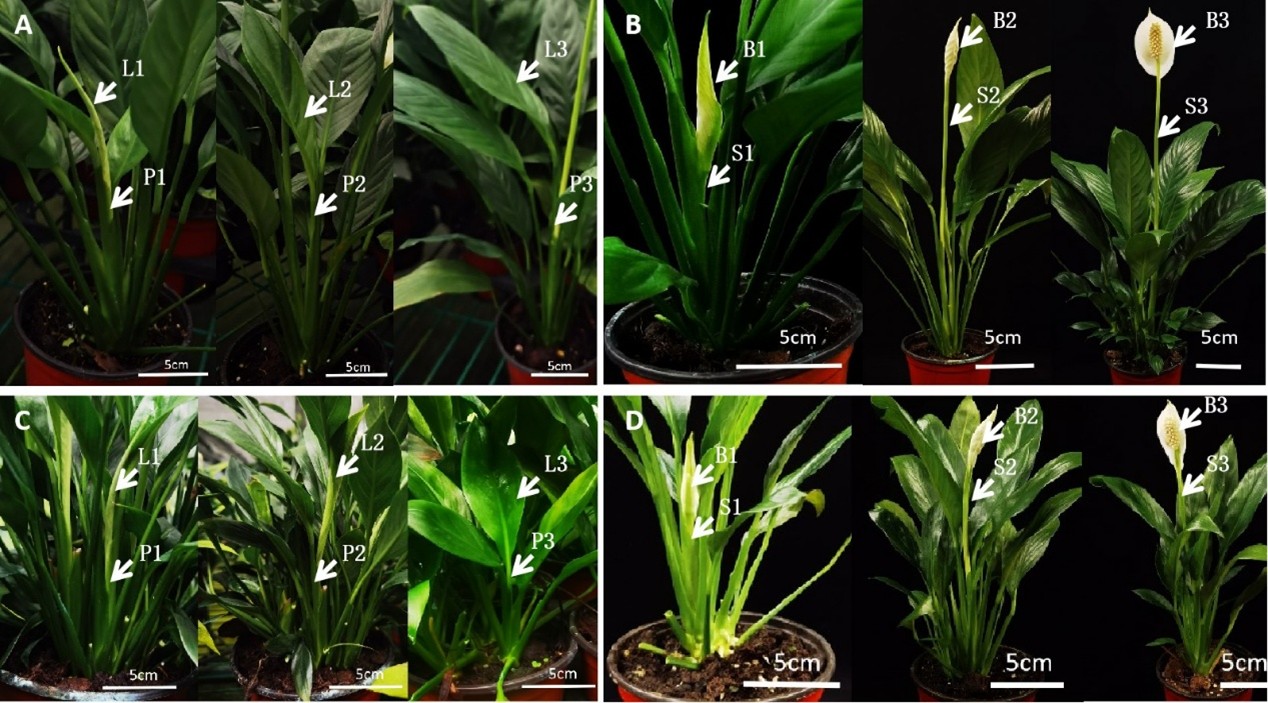


**Supplementary Figure 1.** Morphological comparison and tissue sampling of *S. kochii* cultivars ‘Meijiu’ and ‘Meibian’. (A) Representative images showing three developmental stages of leaf (L1–L3) and petiole (P1–P3) of ‘Meijiu’. (B) Representative images showing three developmental stages of spathe (B1–B3) and spadix (S1–S3) of ‘Meijiu’. (C) Representative images of leaf (L1–L3) and petiole (P1–P3) stages of ‘Meibian’. (D) Representative spathe (B1–B3) and spadix (S1–S3) stages of ‘Meibian’. All samples were collected at the initial flowering stage under uniform growth conditions. White arrows indicate specific tissues sampled for transcriptomic analysis. Scale bars = 5 cm.


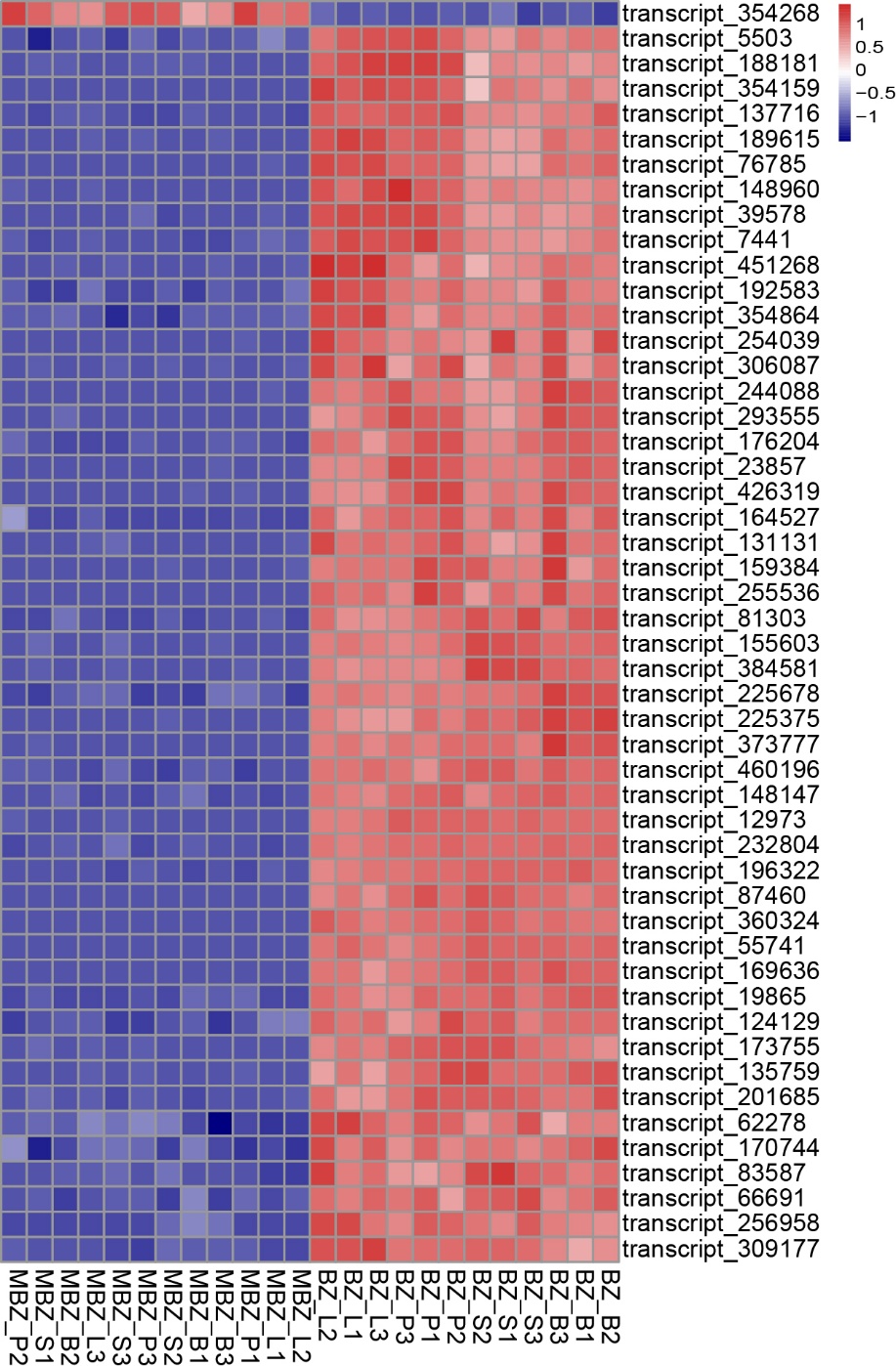
**Supplementary Figure 2.** Heatmap of the top 50 hub genes in module M3 identified by WGCNA. Each row represents a transcript, and each column represents a biological replicate across four tissues in the cultivar ‘Meijiu’ (BZ) and the dwarf mutant ‘Meibian’ (MBZ), including leaf (L), petiole (P), spathe (B), and spadix (S). Gene expression levels were scaled and Z-score transformed.


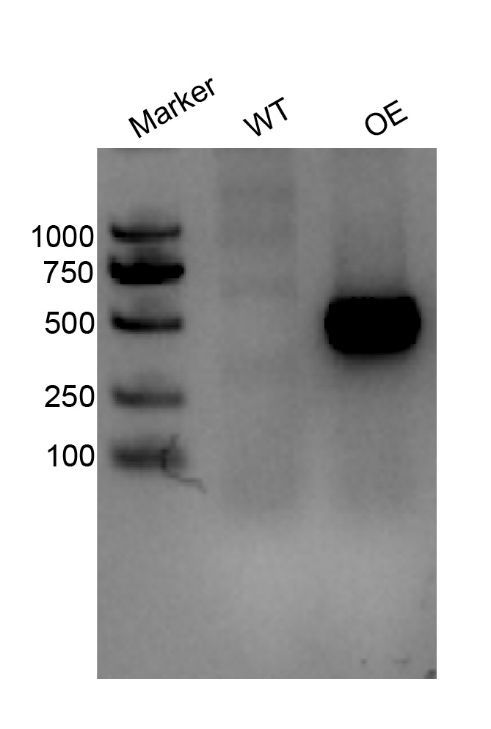


**Supplementary Figure 3.** PCR validation of *SpRCC1* integration in *Arabidopsis thaliana* overexpression lines (OE) compared to wild-type (WT). DNA ladder (Marker) is shown for size reference.

## Supplementary Tables

**Supplementary Table 1.** Primers used in this project.

| Primer | seq(5'-3') |
| --- | --- |
| SpRCC1-F | TCGGGCAATATGCACCATGT |
| SpRCC1-R | CCCTTCCACGTTGAGAGCTT |
| mSpRCC1-F | GGGACGTTCATCATCCAACA |
| mSpRCC1-R | GAGTTTCACAGAAACATCTGCATGT |
| rSpRCC1-R1 | CGCCTTGTTGCCAGCACTAGCTCACCAA |
| rSpRCC1-R2 | CACGACCATGACCAGGAGTTTTCTCCTTGG |
| rSpRCC1-F1 | CCATGAGAGATGGTGCGAAGAGCAGAA |
| rSpRCC1-F2 | GTGGGCGGAAGTTGCATGAGGTCGTAG |
| qSpRCC1-F | CAAAGAATGCTTCAACGGT |
| qSpRCC1-R | CTTTCACCTTCTTGCCAGTT |
| qSpActin-F | TCCTCCTCTTCCTCTTCTTCC |
| qSpActin-R | TTCTTGCACTGGTACACTGG |
| SpRCC1-OE-F | ATTCTCCCTGATTGTTGTCG |
| SpRCC1-OE-R | CTAACTTTCACCTTCTTGCC |

**Supplementary Table 2.** Summary of full-length transcriptome sequencing data.

| Samples | HiFi Reads Number | Full-Length NonChimeric Reads | Full-Length Non-Chimeric Reads with Poly-A Tail |
| --- | --- | --- | --- |
| BZ | 5205006 | 5203368 | 5196120 |
| MBZ | 4876739 | 4875255 | 4869230 |
| total | 10081745 | 10078623 | 10065350 |

**Supplementary Table 3.** Function annotation of *S. kochii*.

**Supplementary Table 4.** Basic stats for RNAseq dataset of 24 libraries.

| sampleID | total_reads | total_bases | Q20 | QC_reads | QC_bases | QC20 |
| --- | --- | --- | --- | --- | --- | --- |
| BZ_B1 | 72517048 | 10877557200 | 0.969871 | 71802316 | 10720068967 | 0.973705 |
| BZ_B2 | 85656650 | 12848497500 | 0.971362 | 84858472 | 12685651954 | 0.97497 |
| BZ_B3 | 63226546 | 9483981900 | 0.973313 | 62679646 | 9359935606 | 0.976843 |
| BZ_L1 | 48153346 | 7223001900 | 0.970966 | 47774164 | 7143145823 | 0.97405 |
| BZ_L2 | 49525100 | 7428765000 | 0.970985 | 49150386 | 7349913770 | 0.973956 |
| BZ_L3 | 42812946 | 6421941900 | 0.973682 | 42509590 | 6355938253 | 0.976509 |
| BZ_P1 | 47818028 | 7172704200 | 0.972894 | 47425670 | 7091694136 | 0.976074 |
| BZ_P2 | 56348216 | 8452232400 | 0.973882 | 55902986 | 8347947471 | 0.977034 |
| BZ_P3 | 44942000 | 6741300000 | 0.971928 | 44560796 | 6654465425 | 0.975188 |
| BZ_S1 | 75860192 | 11379028800 | 0.972304 | 75137688 | 11223150358 | 0.976119 |
| BZ_S2 | 66610694 | 9991604100 | 0.972472 | 65940430 | 9836593454 | 0.976524 |
| BZ_S3 | 58699526 | 8804928900 | 0.971009 | 58058508 | 8665041346 | 0.975348 |
| MBZ_B1 | 48054632 | 7208194800 | 0.970688 | 47638466 | 7122869639 | 0.974086 |
| MBZ_B2 | 58591192 | 8788678800 | 0.973996 | 58143172 | 8689188605 | 0.976977 |
| MBZ_B3 | 48001344 | 7200201600 | 0.973336 | 47609970 | 7114657310 | 0.976687 |
| MBZ_L1 | 53150722 | 7972608300 | 0.971573 | 52652020 | 7856455475 | 0.975261 |
| MBZ_L2 | 53577590 | 8036638500 | 0.973038 | 53162842 | 7945248873 | 0.976158 |
| MBZ_L3 | 52991742 | 7948761300 | 0.971754 | 52566636 | 7857282214 | 0.974889 |
| MBZ_P1 | 48486106 | 7272915900 | 0.974956 | 48133706 | 7192126301 | 0.977957 |
| MBZ_P2 | 49874098 | 7481114700 | 0.971408 | 49473440 | 7397807254 | 0.974548 |
| MBZ_P3 | 55777468 | 8366620200 | 0.973021 | 55347302 | 8268810878 | 0.976081 |
| MBZ_S1 | 51634424 | 7745163600 | 0.975493 | 51236426 | 7661451080 | 0.97867 |
| MBZ_S2 | 42508824 | 6376323600 | 0.967305 | 42078696 | 6282856066 | 0.971183 |
| MBZ_S3 | 42572480 | 6385872000 | 0.973 | 42249200 | 6312602499 | 0.976093 |
